# Seed Yield Components and Seed Quality of Oilseed Rape Are Impacted by Sulfur Fertilization and Its Interactions With Nitrogen Fertilization

**DOI:** 10.3389/fpls.2019.00458

**Published:** 2019-04-16

**Authors:** Emilie Poisson, Jacques Trouverie, S. Brunel-Muguet, Yacine Akmouche, Célia Pontet, Xavier Pinochet, Jean-Christophe Avice

**Affiliations:** ^1^UMR Ecophysiologie Végétale et Agronomie (EVA), Normandie Université, UNICAEN, INRA, SFR Normandie Végétal (FED4277), Caen, France; ^2^Terres Inovia, Centre de Recherche INRA de Toulouse, Bâtiment AGIR, Castanet-Tolosan, France; ^3^Terres Inovia, Direction Etudes et Recherches, Campus INRA Agro ParisTech, Thiverval Grignon, France

**Keywords:** *Brassica napus* L., fertilization, sulfur deficiency, sulfur/nitrogen interactions, seed yield, seed quality, protein quality

## Abstract

Although the impact of sulfur (S) availability on the seed yield and nutritional quality of seeds has been demonstrated, its impact coupled with nitrogen (N) availability remains poorly studied in oilseed rape. A deeper knowledge of S and N interactions on seed yield components and seed quality could improve S and N fertilization management in a sustainable manner. To address this question, our goals were to determine the effects of nine different S fertilization management strategies (i) in interaction with different levels of N fertilization and (ii) according to the timing of application (by delaying and fractionating the S inputs) on agronomic performances and components of seed yield. The impact of these various managements of S and N fertilizations was also investigated on the seed quality with a focus on the composition of SSPs (mainly represented by napins and cruciferins). Our results highlighted synergetic effects on S and N use efficiencies at optimum rates of S and N inputs and antagonistic effects at excessive rates of one of the two elements. The data indicated that adjustment of S and N fertilization may lead to high seed yield and seed protein quality in a sustainable manner, especially in the context of reductions in N inputs. Delaying S inputs improved the seed protein quality by significantly increasing the relative abundance of napin (a SSP rich in S-containing amino acids) and decreasing the level of a cruciferin at 30 kDa (a SSP with low content of S-amino acids). These observations suggest that fractionated or delayed S fertilizer inputs could provide additional insights into the development of N and S management strategies to maintain or improve seed yield and protein quality. Our results also demonstrated that the S% in seeds and the napin:30 kDa-cruciferin ratio are highly dependent on S/N fertilization in relation to S supply. In addition, we observed a strong relationship between S% in seeds and the abundance of napin as well as the napin:30 kDa-cruciferin ratio, suggesting that S% may be used as a relevant index for the determination of protein quality in seeds in terms of S-containing amino acids.

## Introduction

Sulfur (S) is an essential element for growth and metabolic functioning in plants (Leustek and Saito, 1999). Like nitrogen (N), S is an important constituent of proteins due to S-containing essential amino acids like methionine as well as non-essential amino acids like cysteine in particular, which allows the formation of disulfide bonds for protein structure and function (Brosnan and Brosnan, 2006). S fertilization in crops has been of concern since the 1980s as a result of environmental policies that aimed to reduce atmospheric sulfur dioxide (SO_2_) from industrial emissions (Schnug et al., 1993; McGrath and Zhao, 1996; McGrath et al., 2002). As a consequence, S deposition into the soil was strongly reduced (McNeill et al., 2005) leading to increasing occurrence of S deficiency in crops, mainly in Western Europe. Oilseed rape (*Brassica napus* L.) is a high S-demanding crop because of its high contents in sulfate and S-containing secondary metabolites compared to other species like wheat (Oenema and Postma, 2003). Thus, S limitation can severely impact seed yield (between 40 and 50% of loss) and quality in oilseed rape (Zhao et al., 1997). In order to prevent S deficiency, the recommendations from the technical center for oilseed production in France (Terres Inovia) are to provide about 30 kg of S ha^-1^ once at the bolting stage (GS32, Lancashire et al., 1991). However, S-inputs can range from 15 to 60 kg S ha^-1^ depending on the environment (soil type, previous crops, etc.) (Grant et al., 2012). Thus, it is difficult to recommend adequate S inputs because of the lack of indicators of soil and/or plant S status that can be used easily in the field.

Seeds of oilseed rape are an important source of oil and proteins for diverse nutritional and non-edible uses. As proteins of grain legumes (Galili et al., 2005; Krishnan, 2005; Taylor et al., 2008), proteins accumulated in seeds of oilseed rape contain high level of S-amino acids which are essential in meal used for feeding livestock. These seed proteins with high level of S-containing amino acids could be potentially used in human food products with the increasing global demands for vegetable proteins for human nutrition (vegetarian or vegan diets) (Von Der Haar et al., 2014). Moreover, oilseed rape protein isolate has been suggested as an alternative to other proteins for human food use due to a balanced amino acid profile and potential functional properties such as emulsifying, foaming, and gelling abilities (Tan et al., 2011; Campbell et al., 2016; Mupondwa et al., 2018). In addition, antioxidant, antidiabetic, anorexigenic, anticancer, antiviral, hypercholesterolemic, and bile acid binding activities have been reported for peptides and hydrolysate fractions generated from seed proteins of oilseed rape (Wanasundara, 2011; Aachary and Thiyam, 2012).

Previous studies have demonstrated the tight relationship between plant S status or sulfate availability in the soil and the quality of oil and protein in oilseed rape (Zhao et al., 1997; Dubousset et al., 2010; D’Hooghe et al., 2014). Protein quality might be determined by the level of S-containing amino acids, which impacts the levels of S-rich or S-poor SSPs. Indeed, compared to protein isolate from soybean or casein, which contain 0.97% and 2.6% of S-containing amino acids (methionine+cysteine), respectively (Wang et al., 2008), protein isolate from oilseed rape meal have at least 2.99% of S-containing amino acids. This amount exceeded the requirement of FAO/WHO (1985) for children and adults. Cruciferins (11–12S globulins) are the major form of SSP present in Brassica species and account for about 50% of the total seed protein content (Bérot et al., 2005). Napins (2S albumin) represent 10–20% of the SSPs and are very rich in S-containing amino acids (10% of the total protein amino acids) (Monsalve et al., 1991). Thus, limitation of S fertilization leads to decreased seed protein content and/or favors the accumulation of S-poor SSPs (as cruciferin CruBnC1) to the detriment of S-rich SSPs such as napin and cruciferin of CRU4 type (Higashi et al., 2006; D’Hooghe et al., 2014).

A large number of studies have focused on the impact of N on seed yield and its components (Allen and Morgan, 1972) with the perspective of reducing N fertilizer inputs because of deleterious environmental impacts that drastically increased following the boom of the Green Revolution in the 1970s (Davies, 2003). Despite its high requirements of N fertilizers (160–250 kg ha^-1^), oilseed rape is a crop characterized by a weak NUE because only 50% of the N fertilizers is recovered in seeds (Schjoerring et al., 1995). Similarly, the SHI (expressed as S amount in seeds divided by total S in the whole crop) is only about 20% in oilseed rape (McGrath and Zhao, 1996; Dubousset et al., 2010), revealing the low SUE in this oleoproteaginous crop. Several works support evidence for strong interactions between S and N metabolism (Karmoker et al., 1991; Koprivova et al., 2000; Hesse et al., 2004; Coleto et al., 2017) particularly for the synthesis of S-containing amino acids. S limitation can decrease NUE (Schnug et al., 1993; Fismes et al., 2000) and N deficiency can also reduce SUE (Fismes et al., 2000; Salvagiotti et al., 2009). Furthermore, the impacts of S limitation vary according to N supply (Janzen and Bettany, 1984) and when one of these elements is low or in excess it can lead to reduced seed yield, growth, and quality of harvested products (Fismes et al., 2000; Malhi and Gill, 2007). The quality criteria targeted in most studies correspond to the seed yield or to the oil and seed protein contents, but less is known about S and N interactions on fatty acids profiles and specific SSPs, which are determining criteria for nutritional quality. Previous studies performed under controlled conditions have shown that an S limitation applied at early flowering (GS53) or at the start of pod filling (GS70) did not affect seed yield and seed protein content but did affect the nutritional quality of the seeds (oil and protein quality) (Dubousset et al., 2010; D’Hooghe et al., 2014). Thus, a decline of oil quality was observed with an increase in the ratio between fatty acids belonging to omega-6 family (mainly represented by linoleic acid, C18:2) and omega-3 family (mainly represented by α-linolenic acid, C18:3). A decrease in the accumulation of S-rich SSPs (like cruciferin CRU4 or napin) compared to S-poor SSP (cruciferin BnC1) was also reported, leading to a decrease in protein quality (D’Hooghe et al., 2014). Although the impact of S availability on the seed nutritional quality has been demonstrated, this impact coupled with N availability remains poorly studied. A deeper knowledge of the effect of S and N interactions on seed yield components and seed quality could improve S and N fertilization management either by adjusting the amount of fertilizer or the timing of applications. In the case of N fertilization, applications may occur once or be split across two to four inputs during the growth cycle (source Terres Inovia). With the same idea, testing new technical routes/schedules to fractionate or delay S applications might also be worth exploring in order to better respond to plant needs throughout the crop cycle.

Therefore, the aims of this study were to determine the effects of different S fertilization management strategies (i) on interactions with different levels of N fertilization and (ii) according to the timing of application, which includes assessing the impact of delaying and fractionating conditions on seed yield and seed nutritional quality in oilseed rape. Outcomes of this analysis will target the definition of innovative S fertilization strategies in terms of application (timing and fractionating designs) that consider S×N interactions and also the determination of S related indicators of seed quality.

## Materials and Methods

### Experimental Treatments and Tissue Sampling

**FIGURE 1:**
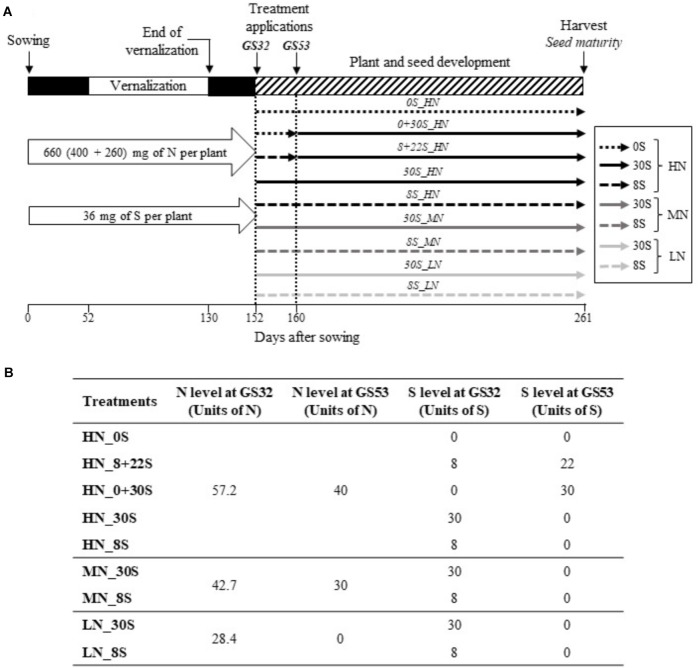
Schematic diagram of experimental design **(A)** and table of the different S and N fertilization strategies **(B)**. Seven combinations of S and N fertilization treatments with S levels of 0 kg S ha^-1^ (0S), 8 kg S ha^-1^ (8S), and 30 kg S ha^-1^ (30S), and N levels from 28.4 kg N ha^-1^ (Low N, LN), 72.7 kg N ha^-1^ (Mid N, MN), and 97.2 kg N ha^-1^ (High N, HN), which were applied manually to the plants. N supplies were provided at the bolting (GS32) and early flowering stages (GS53). S supplies (30S and 8S) were provided at the GS32 stage and were included with each N condition. The 0S condition was only included with the HN condition (negative control). To investigate the impact on seed nutritional quality of delaying or fractionating S inputs, S was provided at GS32 and/or at GS53 under non-limiting N conditions (HN). The fractionated condition (8+22_HN) corresponded to a dual input of 8U and 22U of S provided at GS32 and GS53, respectively. The delayed S input (0+30S_HN) corresponded to a single input of 30 U of S provided at GS53.

The experimental design is described in Figure 1A. Briefly, sterilized seeds of *B. napus* L. (cv. Aviso) were germinated on vermiculite and were grown initially under greenhouse conditions with a thermoperiod of 20°C (day-16 h) and 15°C (night-8 h) for 26 days. Natural light was supplied by high-pressure sodium lamps (Philips, MASTER GreenPower T400W) with a photosynthetically active radiation (PAR) of 400 μmol photon s^-1^ m^-2^ at the top of the canopy. Plants were supplied with 25% Hoagland nutrient solution (0.25 mM KH_2_PO_4_, 1.25 mM KCl, 0.2 mM EDTA, NaFe, 3H_2_O, 14 μM H_3_BO_3_, 5 μM MnSO_4_,7H_2_O, 3 μM ZnSO_4_,7H_2_O, 0.7 μM (NH_4_)_6_Mo_7_O_24_, 0.1 μM CoCl_2_, 0.04 μM NiCl_2_) renewed twice a week. Each plant also received a total of 36 mg of S and 400 mg of N, provided at three different times before vernalization (18, 28, and 40 days after sowing) by a solution of MgSO_4_,7H_2_O and Ca(NO_3_)_2_,4H_2_O (Figure 1A). Twenty-six days after sowing, plants were transferred into 2 L pots containing mixed vermiculite (1V) and perlite (2V). Plants were then submitted to a vernalization period for 78 days: 46 days under natural outdoor conditions (temperature between 2 and 14°C and photoperiod from 8 to 9 h) followed by 32 days under controlled conditions at 8°C (day-10 h) and 4°C (night-14 h). After vernalization, plants were submitted to a thermoperiod of 20°C (day-16 h) and 15°C (night-8 h) and transferred to a greenhouse. As the first N input after vernalization, each plant was supplied with 0.26 g of N with a solution of Ca(NO_3_)_2_,4H_2_O, corresponding to an equivalent of 100 Units of N calculated for a plant density of 40 plants per square meter (100 U: 100 kg N ha^-1^; Figure 1A,B).

In order to mimic S and N inputs provided under field conditions, seven combinations of S and N fertilization treatments with levels of S including 0 kg S ha^-1^ (0S), 8 kg S ha^-1^ (8S), and 30 kg S ha^-1^ (30S), and levels of N including 28.4 kg N ha^-1^ (Low N, LN), 72.7 kg N ha^-1^ (Mid N, MN), and 97.2 kg N ha^-1^ (High N, HN) were applied to the plants manually (Figure 1A,B). N supplies were provided at the bolting stage (GS32; 57.2, 42.7, or 28.4 units of N; Lancashire et al., 1991) and at the early flowering stage (GS53; 40, 30, or 0 units of N) for HN, MN, and LN conditions, respectively. S supplies (30S or 8S) were provided at the GS32 stage and were associated with each N condition. The 0S condition was only associated with the HN condition (negative control). Plants were grown until the production of mature seeds (GS99, final harvest).

The second aim of this work was to investigate the impact of delaying or fractionating S inputs on seed nutritional quality. In non-limiting N conditions (HN), S was provided at one or two different stages of development: at GS32 and/or at GS53. The fractionated condition (8+22_HN) corresponded to an input of 8 and 22 units of S provided at GS32 and GS53, respectively. The delayed S input (0+30S_HN) corresponded to a single input of 30 units of S provided at GS53 and the 30+0S_HN treatment corresponded to a single input of 30 units of S provided at GS32 (control treatment corresponding to the 30S_HN condition, Figure 1B). Plants were grown until production of mature seeds.

For each harvest date, the different plant parts (roots, leaves, stem, inflorescences + immature pods, pod walls, and seeds) were weighed before (fresh matter) and after (DM) freeze-drying and then ground using the system Retsch MM200 (Eragny sur Oise, France) to fine powder for elemental analyses. After freeze-drying, the seeds were stored at -20°C for protein analysis. The seed yield components (total seed weight per plant, thousand seed weight) and the HI (fraction of the total DM allocated to the seed) were also determined.

### S and N Analyses and Determination of SHI, NHI, SUE, and NUE

Freeze-dried and ground plant parts were weighed and placed into tin capsules for analysis of both total S and N contents. Total S and N relative concentrations in the different tissues were determined with an elemental analyzer (EA3000, EuroVector, Milan, Italy) connected to a continuous flow isotope mass spectrometer (IRMS, Isoprime, GV Instruments, Manchester, United Kingdom). Based on the S and N contents, different indices that indicate the plant N and S status were calculated. First, the SHI and NHI were determined as the S or N amounts in seeds expressed as a percentage of the total amount of S or N in the plant at the final harvest. Secondly, the SUE and NUE were calculated as the seed DM produced per 1 g of S or N provided by fertilizers.

### Determination of Seed Nutritional Quality

#### Oil Content

Intact seeds (about 1–3 g) were placed in a standard ring cup and were scanned on a near infrared monochromator system (FT-NIR MPA, Bruker Corporation, Billerica, MA, United States). The results were determined from an external calibration established for oil content (CRAW, Gembloux, Belgium) and were given as a percentage of oil per seed DM.

#### Extraction and Quantification of Total Proteins in Seeds

Total seed proteins were extracted from 40 mg of seed powder previously ground with liquid nitrogen, as described by Gallardo et al. (2002) (*n* = 5 for 0S_HN, 8S_MN, 30S_MN, 0+30S_HN, and 8+22S_HN, *n* = 4 for 8S_HN, 8S_LN, 30S_HN, and 30S_LN). After 1 h incubation at room temperature in thiourea/urea buffer, the extracts were centrifuged twice at 20,000 ×*g* at 4°C for 10 min. Protein concentration was then determined in the supernatant according to Bradford (1976). Fifty microliters of protein extract was mixed with one volume of Laemmli 2× buffer (Laemmli, 1970) and was heated for 10 min in boiling water. For each extract, 10 μg of proteins was loaded per lane. The SDS–PAGE electrophoresis was carried out on precast stain-free gels (4–15% polyacrylamide gel, Bio-Rad ^1^) in the presence of Tris/SDS/Glycine 2× migration buffer (25 mM/0.1%/192 mM; pH 8.8, Laemmli, 1970). These stain-free gels contain trihalogen compounds that allow reactions with tryptophan residues of proteins that can be detected by fluorescence emission after UV excitation. After electrophoresis (200 volts, 75 mA for 30–40 min), gels were placed on a stain-free tray for the detection of protein bands by fluorescence using the Gel DocTM EZ system ^2^. The level of seed protein abundance was determined by image analysis using Image Lab Software (Bio-Rad ^3^). The mean value of relative abundance for the SSPs at 12 (napin) and 30 kDa (cruciferin) was calculated for each treatment (Supplementary Figure S1).

### Statistical Analyses

The variability of the results is expressed as the mean ± standard error (SE) of *n* replicates (*n* = 4 or 5). Analysis of variance (ANOVA) and the Newman–Keuls mean comparison test were performed with a statistical significance at *p* < 0.05 using Microsoft Excel 2018/XLSTATPremium (Version 15.0, Addinsoft, Inc., Brooklyn, NY, United States). Two-way ANOVAs were performed for S, N, and S×N interaction effects on different variables. S, N, and S×N effects were estimated without considering the 0S_HN condition, which was extreme (and not comparable to an N-deprived condition, which is not compatible with plant growth) and tended to exacerbate the S effect and to silence putative N or S×N effects.

## Results

### Impact of Different Levels of S and N Fertilization

#### Influence of S and N Availability on Biomass Partitioning and Seed Yield

**FIGURE 2:**
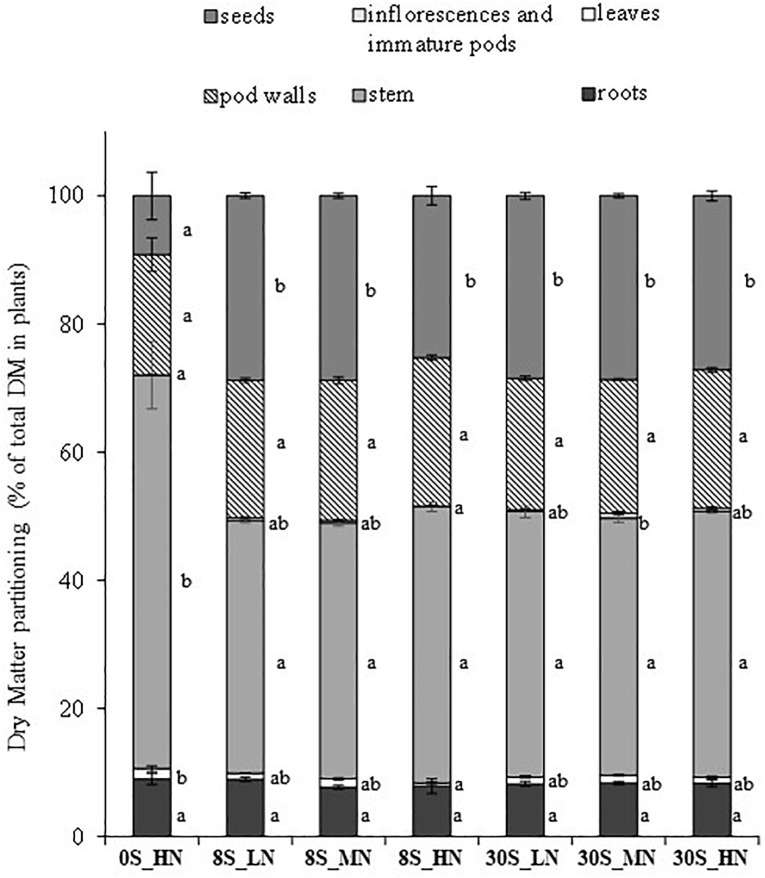
DM partitioning (as % of total DM in plants) for different plant parts (roots, leaves, stems, inflorescence and immature pods, pod walls, and seeds) at harvest for plants grown under 0S_HN, 8S_LN, 8S_MN, 8S_HN, 30S_LN, 30S_MN, and 30S_HN conditions. Vertical bars indicate ± SE of the mean (*n* = 5, *n* = 6 for 8S_LN). Different letters indicate that mean values are significantly different (*p* < 0.05) between treatments for the different plant parts.

Significant S, N, and S×N effects were detected in total plant biomass (Table 1A). Compared to the highest S and N fertilization (30S_HN), the whole plant DM was significantly lower for all the other treatments and was strongly reduced under 0S_HN and 30S_LN conditions (ca. -22.2%), reaching about 28.7 g per plant (Table 1B). The biomass partitioning in 0S_HN strongly differed from the other treatments, especially for stem and seed DM (Figure 2). For instance, stem and seed DM represented, respectively, 61% (17.9 g plant^-1^) and 9% (2.5 g plant^-1^) of the total DM under 0S_HN conditions versus 41% (15.3 g plant^-1^) and 27% (10 g plant^-1^) of the total DM under 30S_HN conditions. The HI was the lowest under 0S_HN conditions (9.2 ± 3.7%). No significant differences in HI were observed between the other treatments (Table 1B). Depending on the environment and genotype, Luo et al. (2015) have reported that HI in oilseed rape ranged from 15 to 36% with an average values between 20 and 27%. In our controlled conditions of culture, except for 0S-HN treatment, we observed an HI between 25.3 and 28.8%, a range of value that is consistent with literature.

**Table 1:** S, N, and S×N effects on total DM, seed DM, thousand seed weight, and HI **(A)** and total DM (g plant^-1^), seed DM (g plant^-1^), thousand seed weight (mg), and HI (DM in seeds as % of total DM in plant) for plants grown under 0S_HN, 8S_LN, 8S_MN, 8S_HN, 30S_LN, 30S_MN, and 30S_HN conditions **(B)**.

A	Treatments	DM_total_	Seed DM	Thousand seed weight	HI
	S effect	^∗∗∗^	^∗∗^	ns	ns
		*p* = 0.0007	*p* = 0.0096	*p* = 0.77	*p* = 0.44
	N effect	^∗∗^	ns	^∗∗^	^∗∗^
		*p* = 0.003	*p* = 0.158	*p* = 0.004	*p* = 0.003
	S×N effect	^∗∗^	^∗∗^	ns	ns
		*p* = 0.006	*p* = 0.0031	*p* = 0.16	*p* = 0.28

**B**	**Treatments**	**DM_total_ (g ⋅ plant^-1^)**	**Seed DM (g ⋅ plant^-1^)**	**Thousand seed weight (mg)**	**HI (DM in seeds as % of total plant DM)**

	0S_HN	28.8 ± 1.0^a^	2.5 ± 1.0^a^	2971 ± 693^a^	9.2 ± 3.7^a^
	8S_LN	29.7 ± 1.0^a^	8.5 ± 0.3^bc^	3350 ± 134^a^	28.8 ± 0.4^b^
	8S_MN	30.7 ± 0.8^a^	8.8 ± 0.2^bc^	3138 ± 124^a^	28.8 ± 0.4^b^
	8S_HN	30.2 ± 1.1^a^	7.6 ± 0.5^b^	3434 ± 47^a^	25.3 ± 1.5^b^
	30S_LN	28.7 ± 0.7^a^	8.2 ± 0.2^bc^	3211 ± 55^a^	28.5 ± 0.5^b^
	30S_MN	33.1 ± 1.3^a^	9.5 ± 0.3^bc^	3220 ± 348^a^	28.6 ± 0.3^b^
	30S_HN	37.0 ± 1.7^b^	10.1 ± 0.6^c^	3578 ± 168^a^	27.2 ± 0.8^b^


*S, N, and S×N effects were estimated by omitting 0S_HN condition. ^∗^*p* < 0.05; ^∗∗^*p* < 0.01; ^∗∗∗^*p* < 0.001; ns, not significant **(A)**. The values correspond to the mean ± SE (*n* = 5, *n* = 6 for 8S_LN). Different letters indicate that mean values are significantly different (*p* < 0.05) (**B**).*

For seed yield, S and S×N effects were detected but no significant N effect was observed (Table 1A). The highest seed yield was reached under 30S_HN conditions (10.1 g plant^-1^) while the lowest seed yield was observed for the 0S_HN treatment (2.5 g plant^-1^, Table 1B). The seed yield under 8S_HN, which was one of the most unbalanced S and N treatments, was significantly different from 30S_HN conditions. For the thousand seed weight, only N effect was detected (Table 1A), with the highest values observed under HN for a given S supply (Table 1B). This means that under HN conditions, seed number was lower under 8S_HN than under 30S_HN.

#### Effects of Treatments on S and N Partitioning

**FIGURE 3:**
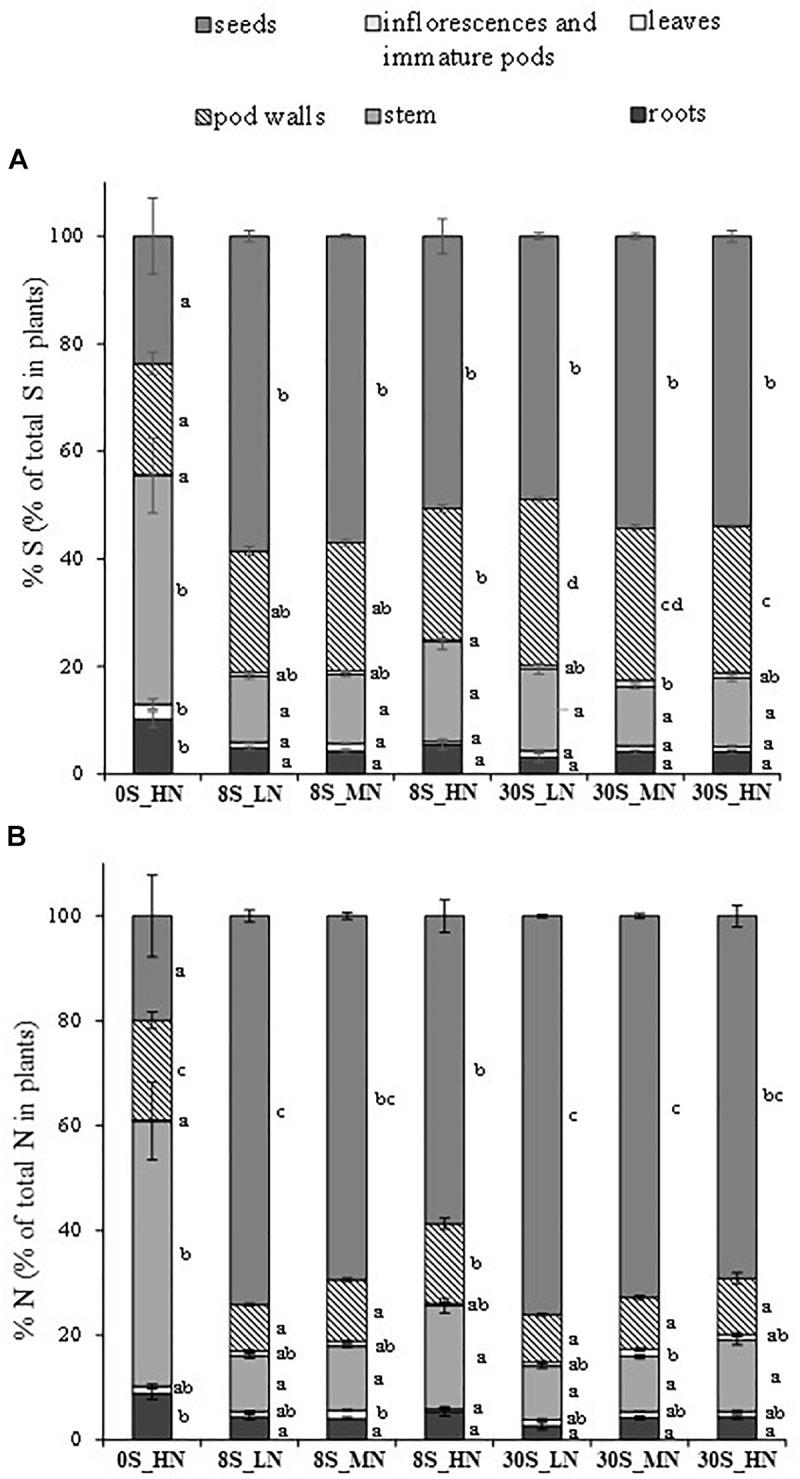
Final S partitioning (%) **(A)** and final N partitioning (%) **(B)** for plants grown under 0S_HN, 8S_LN, 8S_MN, 8S_HN, 30S_LN, 30S_MN, and 30S_HN conditions. Vertical bars indicate ± SE of the mean (*n* = 5, *n* = 6 for 8S_LN). Different letters indicate that mean values are significantly different (*p* < 0.05) between treatments for the different plant parts.

Sulfur and N effects were detected in the total S amount (Table 2A). As expected, the total S amount decreased as a function of the reduction in S supply, for instance, from 89.5 mg per plant under 30S_HN conditions to 27.7 mg of S per plant under 0S_HN (Table 2B). Out of all the treatments, 0S_HN had the greatest effect on the final S partitioning among the different plant parts (Figure 3A). The majority of the S in plants grown under the 0S_HN treatment was allocated to the stem (42.5% of the total S in the plant) while the larger proportion of S was allocated toward the seeds in the other treatments (48.9–58.5% of the total S in the plants). The proportion of S in pod walls was significantly reduced by S limitation (0S_HN and all of the 8S conditions) and reached on average 23% of the total S in the plants, versus 29% under the 30S conditions. Under 0S_HN conditions, the proportion of S in roots (10.2%) was also significantly higher than the other treatments where proportion of S ranged from 3 to 5.5%. The distribution of S under the different conditions was less contrasting for the leaves (significantly higher for 0S_HN) and for the inflorescence and immature pods (significantly lower for 0S_HN and 8S_HN). Significant S and S×N effects were observed in the SHI (Table 2A). The lowest SHI was observed for the 0S_HN treatment with 23.7% of the total S recovered in the seeds (Table 2B and Figure 3A).

**Table 2:** S, N, and S×N effects on total N in plants, total S in plants, the NHI, the SHI, N use efficiency (NUE), and S use efficiency (SUE) **(A)** and total N in plants (mg plant^-1^), total S in plants (mg plant^-1^), the NHI (N in seeds as % of total N in plants), the SHI (S in seeds as % of total S in plants), NUE (g of mature seed DM per g of N input), and SUE (g of mature seed DM per g of S input) for plants grown under 0S_HN, 8S_LN, 8S_MN, 8S_HN, 30S_LN, 30S_MN, and 30S_HN conditions **(B)**.

A	Treatments	Total S in plants	Total N in plants	SHI	NHI	SUE	NUE
	S effect	^∗∗∗^	^∗∗∗^	^∗^	^∗∗∗^	^∗∗∗^	^∗^
		*p* < 0.0001	*p* = 0.0002	*p* = 0.012	*p* = 0.0006	*p* < 0.0001	*p* = 0.015
	N effect	^∗∗^	^∗∗∗^	ns	^∗∗∗^	ns	^∗∗^
		*p* = 0.0012	*p* < 0.0001	*p* = 0.095	*p* < 0.0001	*p* = 0.206	*p* = 0.0026
	S×N effect	ns	^∗∗∗^	^∗∗∗^	^∗^	^∗∗^	^∗∗^
		*p* = 0.145	*p* = 0.0006	*p* = 0.00064	*p* = 0.031	*p* = 0.006	*p* = 0.0039

**B**	**Treatments**	**Total S in plants (mg ⋅ plant^-1^)**	**Total N in plants (mg ⋅ plant^-1^)**	**SHI (S in seeds as % of total S in plants)**	**NHI (N in seeds as % of total N in plants)**	**SUE (g of mature seed DM/g of S input)**	**NUE (g of mature seed DM/g of N input)**

	0S_HN	27.7 ± 3.3^a^	543 ± 13.8^bc^	23.7 ± 7.1^a^	19.9 ± 7.8^a^	70.7 ± 28.1^a^	2.8 ± 1.1^a^
	8S_LN	66.6 ± 2.5^c^	491 ± 15.6^a^	58.5 ± 1.0^b^	74.2 ± 1.1^c^	152.6 ± 5.6^b^	11.7 ± 0.4^c^
	8S_MN	59.3 ± 3.1^c^	522 ± 10.1^ab^	57.0 ± 0.4^b^	69.4 ± 0.7^bc^	158.0 ± 3.4^b^	10.4 ± 0.2^c^
	8S_HN	45.5 ± 4.4^b^	577 ± 2.9^c^	50.6 ± 3.2^b^	58.7 ± 3.2^b^	135.7 ± 8.6^b^	8.4 ± 0.5^b^
	30S_LN	96.6 ± 3.4^d^	483 ± 6.4^a^	48.9 ± 0.6^b^	76.1 ± 0.3^c^	77.1 ± 2.2^a^	11.2 ± 0.2^c^
	30S_MN	101.1 ± 3.6^d^	560 ± 12.4^bc^	54.3 ± 0.5^b^	72.8 ± 0.5^c^	89.3 ± 3.3^a^	11.1 ± 0.4^c^
	30S_HN	89.5 ± 5.1^d^	669 ± 11.6^d^	53.9 ± 1.1^b^	69.1 ± 2.1^bc^	94.9 ± 5.8^a^	11.2 ± 0.7^c^


*S, N, and S×N effects were estimated by omitting 0S_HN condition. ^∗^*p* < 0.05; ^∗∗^*p* < 0.01; ^∗∗∗^*p* < 0.001; ns, not significant **(A)**. The values correspond to the mean ± SE (*n* = 5, *n* = 6 for 8S_LN). Different letters indicate that mean values are significantly different (*p* < 0.05) **(B)**.*

Highly significant S, N, and S×N effects were detected in the total N amount in plants at the final stage of harvest (Table 2A). As expected, the total N amount in plants was strongly reduced by LN treatment, whatever the S supply (483 mg plant^-1^ for 30S_LN), when compared to 30S_HN conditions (669 mg plant^-1^) (Table 2B). The distribution of N in plants was very different under 0S_HN conditions compared to other conditions (Figure 3B) with the highest proportion in stems: 50.6% of the total N versus 10.3% for 30S_LN and 19.7% for 8S_HN. The higher the N supply, the higher the proportion of N in stems under the 8S or 30S treatment (Figure 3B). S, N, and S×N effects were detected in the NHI (Table 2A). In response to S restriction (0S) under HN conditions, the NHI was drastically reduced (around 20% of the total N in plants) (Table 2B). However, a greater NHI was observed under 30S_MN (72.8%), 8S_LN (74.2%), and 30S_LN (76.1%) conditions.

#### Effects of Treatments on S and N Use Efficiencies

Sulfur use efficiency is strongly impacted by S and S×N effects (Table 2A). Indeed, SUE was lower under 0S and 30S than under 8S conditions, whatever the N supply (Table 2B). S, N, and S×N effects were detected between treatments for NUE (Table 2A). Compared to 30S conditions and irrespective of the N supply, NUE was significantly reduced by S deprivation (0S) or S limitation (8S) under non-limiting N conditions (HN) (Table 2B). In the context of S limitation (8S), the increase in N fertilization reduced the NUE by 39.3% (from 11.7 to 8.4 g of mature seed DM per g of N input for 8S_LN and 8S_HN, respectively). However, N supply had no effect on the NUE when S availability was non-limiting, and it reached 11.2 g of mature seed DM per g of N input in 30S_HN conditions.

### Changes in Seed Nutritional Quality in Response to the Management of S and N Fertilization

The relative content of S and N in seeds (%S and %N in DM) was significantly affected by S and N effects (Table 3A). As expected, the lowest %S in seeds was observed under the 0S_HN and 8S-HN treatments while lowest %N in seeds was reported for the treatments 8S-MN, 8S-LN, and 0S-HN (Table 3B). Under 8S conditions, the lower the N supply, the higher the %S in seeds. Similarly, under 30S conditions, the %S in seeds was significantly higher with MN and LN treatments (0.58%) than HN treatments (0.48%) (Table 3B). For oil content, an S effect was observed mainly due to the 0S_HN treatment (with 31.9% of the seed DM, Table 3A). When the 8S and 30S treatments were compared for analysis of the S effect (excluding the 0S_HN treatment so as to avoid distortions), only an N effect was detected in the oil content, and no S or S×N effects were observed. Under 8S conditions, the lower the N supply, the higher the oil content in seeds, which increased by about 6.5% between HN and LN conditions (Table 3B). The protein content in seeds is strongly dependent on the N fertilization effect (Table 3A). Indeed, compared to HN, N limitation (LN) strongly reduced the seed protein content, irrespective of the S fertilization levels, with a reduction of 49.3 and 54.5% under 30S and 8S conditions, respectively (Table 3A).

**Table 3:** S, N, and S×N effects on relative content of S, N, oil, and proteins, and on the ratio of napin:30 kDa-cruciferin in mature seeds **(A)** and S, N contents (% of DM), oil content (in % of DM, estimated by NIRS), protein content (mg g^-1^ DM), and the napin:30 kDa-cruciferin ratio in mature seeds for plants grown under 0S_HN, 8S_LN, 8S_MN, 8S_HN, 30S_LN, 30S_MN, and 30S_HN conditions **(B)**.

	Treatments	S in mature seeds (% DM)	N in mature seeds (% DM)	Oil content in mature seeds (% DM)	Protein content in mature seeds (mg ⋅ g^-1^ DM)	Napin:30 kDa-cruciferin ratio
**A**	S effect	^∗∗∗^	^∗∗^	ns	ns	^∗∗∗^
		*p* < 0.0001	*p* = 0.005	*p* = 0.138	*p* = 0.069	*p* < 0.0001
	N effect	^∗∗∗^	^∗^	^∗∗^	^∗∗∗^	^∗∗∗^
		*p* < 0.0001	*p* = 0.011	*p* = 0.007	*p* < 0.0001	*p* < 0.0001
	S×N effect	ns	ns	ns	ns	^∗^
		*p* = 0.152	*p* = 0.886	*p* = 0.066	*p* = 0.393	*p* = 0.012

**B**	0S_HN	0.288 ± 0.007^a^	4.20 ± 0.04^ab^	31.9 ± 1.0^a^	105 ± 5.2^b^	0.1 ± 0.03^a^
	8S_LN	0.464 ± 0.026^c^	4.29 ± 0.09^ab^	39.4 ± 0.4^c^	77 ± 9.2^a^	1.0 ± 0.27^bcd^
	8S_MN	0.384 ± 0.025^b^	4.09 ± 0.05^a^	38.6 ± 0.4^bc^	118 ± 1.5^b^	0.7 ± 0.12^abc^
	8S_HN	0.302 ± 0.026^a^	4.47 ± 0.09^bc^	36.9 ± 0.5^b^	119 ± 13.3^b^	0.4 ± 0.07^ab^
	30S_LN	0.578 ± 0.015^d^	4.51 ± 0.09^bc^	39.5 ± 0.5^c^	73 ± 5.2^a^	3.3 ± 0.52^e^
	30S_MN	0.580 ± 0.016^d^	4.32 ± 0.13^abc^	38.0 ± 0.5^bc^	95 ± 3.7^ab^	1.6 ± 0.10^d^
	30S_HN	0.480 ± 0.009^c^	4.63 ± 0.10^c^	38.8 ± 0.5^bc^	109 ± 4.2^b^	1.3 ± 0.23^cd^


*S, N, and S×N effects were estimated by omitting 0S_HN condition. ^∗^*p* < 0.05; ^∗∗^*p* < 0.01; ^∗∗∗^*p* < 0.001; ns, not significant **(A)**. The values correspond to the mean ± SE (for oil content, *n* = 5 and *n* = 6 for 8S_LN; for protein content, *n* = 5; for napin/cruciferin, *n* = 5 for 0S_HN, 8S_MN, and 30S_MN and *n* = 4 for 8S_HN, 8S_LN, 30S_HN, and 30S_LN). Different letters indicate that mean values are significantly different (*p* < 0.05) **(B)**.*

**FIGURE 4:**
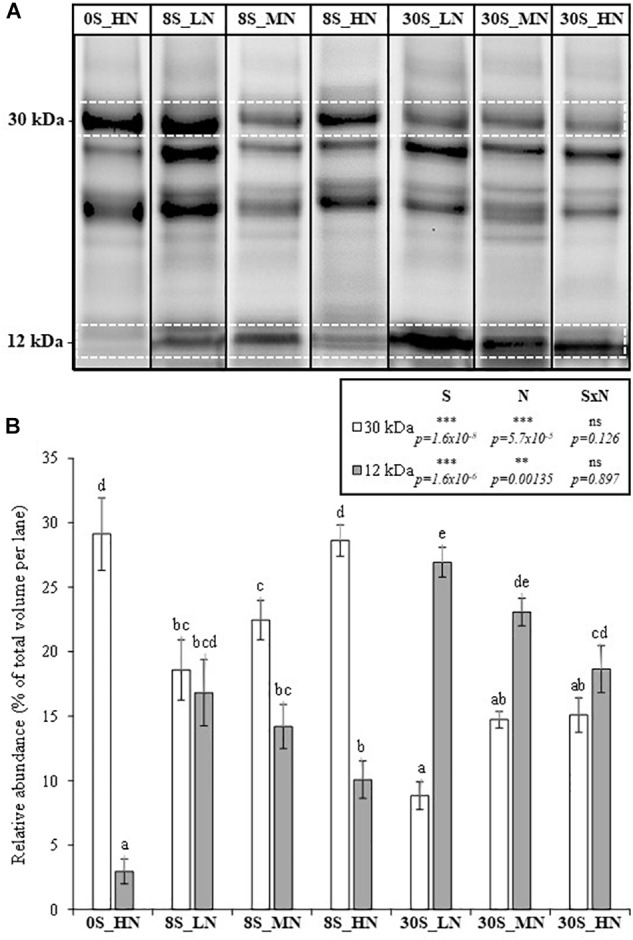
SDS–PAGE of proteomic profiles from mature seeds **(A)** and the relative abundance (% of total volume per lane) of a 30-kDa protein (cruciferin, S-poor protein) and a 12-kDa protein (napin, S-rich protein) in mature seeds **(B)** of plants grown under 0S_HN, 8S_LN, 8S_MN, 8S_HN, 30S_LN, 30S_MN, and 30S_HN conditions. **A**: for a given treatment, the SDS–PAGE was performed with the averaged sample of proteins prepared after mixing the protein extracts from the four or five biological replications. The images of each averaged sample corresponding to each treatment were grouped together in Figure 4A. In **B**: for a given treatment, each biological replication was performed in SDS–PAGE (see Supplementary Data in Supplementary Figure S1) in order to determine the mean value for the relative abundance of proteins at 12 and 30 kDa proteins. Vertical bars indicate ± SE of the mean (*n* = 5 for 0S_HN, 8S_MN, and 30S_MN, *n* = 4 for 8S_HN, 8S_LN, 30S_HN, and 30S_LN). Different letters indicate that mean values are significantly different (*p* < 0.05) between treatments. S, N, and S×N effects were estimated by omitting 0S_HN condition. ^∗^*p* < 0.05; ^∗∗^*p* < 0.01; ^∗∗∗^*p* < 0.001; ns, not significant.

In order to evaluate the seed protein quality, the relative abundances of two different types of SSPs including cruciferin at 30 kDa (S-poor SSP as BnC1 and CRU1) and napin at 12 kDa (S-rich SSP, i.e., which contains higher levels of S-amino acids) were determined after SDS–PAGE on stain-free gels (Figure 4A). A decline in S-rich SSP accumulation was considered as a loss in seed quality. The effects of S and N fertilization on the relative abundances of 30 kDa-cruciferin and napin were significant (Figure 4B). The abundance of napin was drastically reduced under the 0S_HN treatment, comprising only 3% of the total detected seed proteins. Compared to the 0S_HN treatment, the abundance of napin was significantly improved by the increase in S fertilization (3.4- and 6.3-fold higher for 8S_HN and 30S_HN, respectively, Figure 4B). As expected, the relative abundance of napin was significantly higher under 30S than under 8S conditions for a given level of N fertilization (+1.6-fold for LN or MN, +1.9-fold for HN). The opposite occurred for the relative abundance of 30 kDa-cruciferin, which was lower under 30S conditions than under 8S conditions for a given level of N fertilization (Figure 4B). Under non-limiting S conditions (30S), the decrease in the proportion of the 30 kDa-cruciferin associated with high N supplies was significant (Figure 4B). Under 8S conditions, higher N supplies increased the relative proportion of 30 kDa-cruciferin: from 18.6% for 8S_LN to 28.6% for 8S_HN.

In order to provide a reliable indicator of seed protein quality, a ratio between the relative abundance of napin and 30 kDa-cruciferin was calculated (Table 3) and the higher the ratio, the higher the seed quality. The napin:30 kDa-cruciferin ratio was subject to highly significant S, N, and S×N effects (Table 3A). The lowest napin:30 kDa-cruciferin ratio was observed under the 0S_HN treatment (0.1) followed by 8S_HN (0.4). Under 8S conditions, the napin:30 kDa-cruciferin ratio was less than or equal to 1 whereas it was higher than 1 under 30S conditions, reaching 3.63 in the 30S_LN treatment (Table 3B).

### Effects of Fractionated and Delayed S Inputs

The second aim of the study was to investigate the impact of fractionating or delaying S inputs under non-limiting N conditions on yield components, plant N and S status, and seed nutritional quality. Both treatments were compared to the 30+0S_HN treatment (corresponding to the previous 30S_HN).

#### Effects of Fractionated or Delayed S Inputs on Yield Components and S and N Plant Status

**FIGURE 5:**
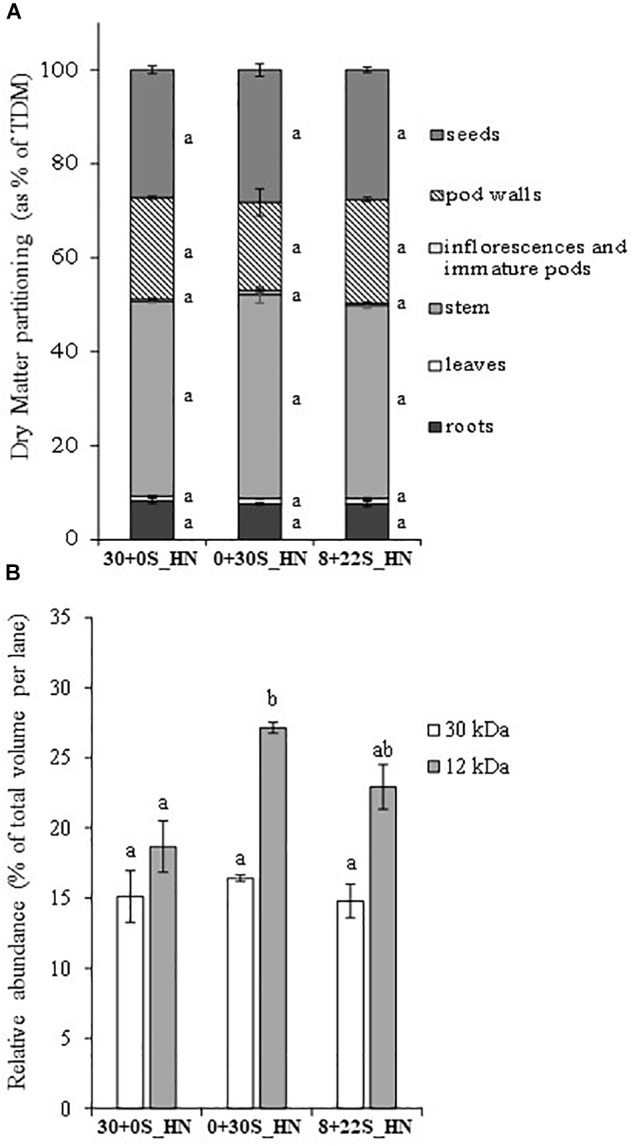
DM partitioning (as % of total DM in plant) **(A)** and relative abundance (% of total volume per lane) of a 30-kDa protein (cruciferin, S-poor protein) and a 12-kDa protein (napin, S-rich protein) in mature seeds **(B)** of plants grown under 30+0S_HN, 0+30S_HN, and 8+22S_HN conditions. Vertical bars indicate ± SE of the mean (*n* = 5, B; *n* = 4 for 30+0S_HN). Different letters indicate that mean values are significantly different.

No significant differences were observed between the treatments for DM partitioning (Figure 5A), total DM, seed DM, thousand seed weight, or HI (Table 4), which meant that fractionated or delayed S inputs had no impact on seed yield components. No significant differences were detected between treatments for the total S in plants, NHI, SHI, NUE, or SUE (Table 5). Concerning N status, the total N amount in plants was significantly higher for plants grown under 30+0S_HN conditions (669 mg plant^-1^) than under 0+30S_HN conditions (603 mg plant^-1^) (Table 5). Plants in the 8+22S_HN treatment had an intermediate total N amount of about 632 mg plant^-1^.

**Table 4:** Total DM (g ⋅ plant^-1^), seed DM (g ⋅ plant^-1^), thousand seed weight (mg), and the HI (DM in seeds as % of total DM in plant) for plants grown under 30+0S_HN, 0+30S_HN, and 8+22S_HN conditions.

Treatments	DM_total_ (g ⋅ plant^-1^)	Seed DM (g ⋅ plant^-1^)	Thousand seed weight (mg)	HI (DM in seeds as % of total DM in plants)
30+0S_HN	37.0 ± 1.7^a^	10.1 ± 0.4^a^	3578 ± 75^a^	27.2 ± 0.8^a^
0+30S_HN	35.8 ± 1.1^a^	10.1 ± 0.6^a^	3584 ± 43^a^	28.2 ± 1.4^a^
8+22S_HN	36.6 ± 0.7^a^	10.1 ± 0.3^a^	3541 ± 92^a^	27.6 ± 0.5^a^


*The values correspond to the mean ± SE (*n* = 5). Different letters indicate that mean values are significantly different (*p* < 0.05).*

**Table 5:** Total N in plants (mg plant^-1^), total S in plants (mg plant^-1^), NHI (N in seeds as % of total N in plant), SHI (S in seeds as % of total S in plant), N use Efficiency (NUE; g of mature seed DM/g of N input), and S use Efficiency (SUE; g of mature seed DM/g of S input) for plants grown under 30+0S_HN, 0+30S_HN, and 8+22S_HN conditions.

Treatments	Total S in plants (mg ⋅ plant^-1^)	Total N in plants (mg ⋅ plant^-1^)	SHI (S in seeds as % of total S in plants)	NHI (N in seeds as % of total N in plants)	SUE (g of mature seed DM/g of S input)	NUE (g of mature seed DM/g of N input)
30+0S_HN	89.5 ± 5.1^a^	669 ± 11.6^b^	53.9 ± 1.1^a^	69.1 ± 2.1^a^	94.9 ± 5.8^a^	11.2 ± 0.7^a^
0+30S_HN	91.1 ± 3.5^a^	603 ± 10.8^a^	56.9 ± 2.8^a^	67.7 ± 2.3^a^	94.8 ± 3.9^a^	11.2 ± 0.5^a^
8+22S_HN	98.3 ± 4.4^a^	632 ± 24.4^ab^	53.6 ± 1.8^a^	69.3 ± 1.6^a^	95.4 ± 2.7^a^	11.2 ± 0.3^a^


*The values correspond to the mean ± SE (*n* = 5). Different letters indicate that mean values are significantly different (*p* < 0.05).*

#### Effects of Fractionated or Delayed S Supply on Seed Nutritional Quality

Delaying or fractionating the S supply did not significantly affect %S as well as oil and protein contents in seeds (Table 6). Delaying the S supply significantly reduced the %N in seeds (Table 6). The relative abundance of napin (12 kDa, S-rich SSP) and 30 kDa-cruciferin (S-poor SSP) (Figure 5B) revealed a significant increase in the percentage of napin under 0+30S_HN conditions (27.1%) compared to 30+0S_HN conditions (18.7%), with an intermediate level observed under 8+22S_HN conditions (22.9%). The napin:30 kDa-cruciferin ratio presented in Table 6 showed no significant differences between the three treatments. It was higher than 1 and ranked as 1.30, 1.66, and 1.70 for 30+0S_HN, 0+30S_HN, and 8+22S_HN treatments, respectively.

**Table 6:** Relative content of S, N and oil (in % of DM), protein content (mg g^-1^ DM), and the napin:30 kDa-cruciferin ratio in mature seeds for plants grown under 30+0S_HN, 0+30S_HN, and 8+22S_HN conditions.

Treatments	S in mature seeds (% DM)	N in mature seeds (% DM)	Oil content in mature seeds (% DM)	Protein content in mature seeds (mg ⋅ g^-1^ DM)	Napin:30 kDa-cruciferin ratio
30+0S_HN	0.480 ± 0.009^a^	4.63 ± 0.10^b^	38.8 ± 0.5^a^	109 ± 4.2^a^	1.30 ± 0.23^a^
0+30S_HN	0.514 ± 0.012^a^	4.07 ± 0.06^a^	38.6 ± 0.5^a^	104 ± 5.2^a^	1.66 ± 0.04^a^
8+22S_HN	0.520 ± 0.019^a^	4.34 ± 0.16^ab^	39.4 ± 0.5^a^	96 ± 9.1^a^	1.70 ± 0.13^a^


*The values correspond to the mean ± SE (*n* = 5, *n* = 4 for napin/cruciferin ratio for 30+0S_HN). Different letters indicate that mean values are significantly different (*p* < 0.05).*

## Discussion

### Adjustment of S and N Inputs to Optimize Growth, Seed Yield, and S and N Use Efficiencies

The results of our study are in line with previous studies in Brassicaceae species (Koralewska et al., 2007; Dubousset et al., 2009) which reported that biomass allocation was strongly affected under severe S limitation (Figure 2). As expected, total DM and seed DM were significantly reduced by 0S_HN treatment (Scherer, 2001) as well as SUE, NUE, SHI, and NHI (Table 2). Under 0S_HN conditions, it appears that stem contains more S (10.97 mg of S ± 1.04) than 8S_LN, 8S-MN, and 8S_HN where stem contains 8.14 ± 0.43, 7.62 ± 0.46, and 8.32 ± 0.48 mg of S, respectively (data not shown). Under 0S_HN conditions, N accumulation is also significantly more important in stem (276.41 mg of N ± 43.11) when compared to other treatments where N in stem ranged from 49.74 mg of N in 30S_LN to 114.05 mg of N in 8S_HN (data not shown). Thus, as indicated in Figure 3 and according with SHI and NHI (Table 1), these data confirm that the stem of 0S_HN tends to accumulate S and N, suggesting that S and N were sequestered mainly in the stem, which was a higher sink organ (Figure 3). Furthermore, the analysis of the effects of various S and N supplies on plant growth, DM allocation, and S and N plant status highlighted the tight interaction between S and N metabolism (Fismes et al., 2000; Kopriva and Rennenberg, 2004; Salvagiotti and Miralles, 2008; Anjum et al., 2012) and the necessity to jointly monitor S and N fertilization. The increase in S fertilization in non-limiting N conditions led to improvement in the seed yield (from 7.6 to 10 g plant^-1^), whereas it had no effect on seed yield under N limiting conditions. Dubousset et al. (2010) showed that low S conditions improved S remobilization into the sulfate form from the leaves independently of N during the grain filling period in order to satisfy seed S requirements. This explains the high SUE observed in all the 8S treatments (Table 2). It has been shown in rapeseed that low N availability promoted early N remobilization and recycling (Desclos et al., 2009) and allowed remobilization from the rosette to the seeds. Overall, as expected from other studies (Dubousset et al., 2009; Girondé et al., 2015a,b), N-limiting conditions enhanced the NHI, NUE, and SHI but they interfered with S availability because S×N interaction effects were also detected for the NHI, SHI, NUE, and SUE. Depending on S availability, the response to LN was modulated and was the most impaired under 30S condition, thus showing the importance of balancing the fertilizer N:S ratio carefully. For instance, the NHI increased significantly by 21% between the HN and LN conditions under 8S but was not significantly different under 30S. Our results also highlighted synergetic effects on SUE and NUE at optimum rates of S and N inputs, and antagonistic effects in case of higher rates of one of the two elements, as also described by Fismes et al. (2000). These results clearly indicate that adjustments of S and N fertilizer applications may lead to high seed yields and agronomic performance while increasing sustainability (i.e., a reduction in N fertilizer use).

### Importance of Balancing S and N Inputs to Maintain Seed Nutritional Quality

Previous studies based on proteomic approaches (D’Hooghe et al., 2014) have underlined that seed protein quality was reduced in winter oilseed rape in response to S fertilization limitation. Indeed, S limitation led to reduction in the accumulation of S-rich SSPs such as napin, whereas the accumulation of S-poor SSPs (such as cruciferin BnC1) was favored. In our experiment, although S limitation reduced seed protein quality, seed protein content was not affected by S limitation and remained stable, whatever the level of S fertilization (between 105 and 119 mg g^-1^ seed DM, respectively, Table 3). Accordingly, Malhi and Gill (2007) have reported that higher N inputs increased protein concentration and reduced oil content in oilseed rape seed, whereas S fertilization did not significantly change seed protein concentrations. Our results underlined the negative effect of LN on seed protein content (Table 3), which decreased by 33 and 35% between HN and LN conditions, respectively, under both the 30S and 8S conditions. Moreover, Aminpanah (2013) positively correlated the N rate input fertilizer with the protein content in seed. The present study shows that % of S and oil in seeds was also reduced by a high N input, especially when plants are subjected to S limitation conditions (8S) or more severely by S deprivation (0S) (Table 3). Our results confirmed that S deprivation (0S_HN) drastically affected the seed protein quality of oilseed rape by reducing the relative abundance of napin, without strong reductions in seed protein content (D’Hooghe et al., 2014). In contrast, the relative abundance of 30 kDa-cruciferin (S-poor SSP) increased, which acted as a compensatory mechanism to maintain seed protein content, thus leading to adjustment of the S-rich/S-poor protein ratio. Generally, the relative abundance of napins was lower under 8S than under 30S conditions (Table 2). However, when low S is combined with low N inputs, the napin abundance was not significantly different from the levels observed under high S and high N conditions. This indicated that plants were able to increase their S and N use efficiencies through the optimization of S and N remobilization in response to low S and N availabilities (Table 2). However, despite the high quality of seed protein under the 8S_LN treatment, the protein content remained low. An unexpected effect was the very high relative abundance of napin in seeds of plants grown under 30S_LN conditions, whereas the SHI was low (reached 26.93%). Because S was not limiting and protein content decreased in this condition, the proportion of napins and cruciferins in the seeds was out of balance. The increase in N input fertilizer was positively correlated with the relative abundance of 30 kDa-cruciferin and negatively correlated with the relative abundance of napin under both 8S and 30S conditions.

#### Delaying or Fractioning S Inputs Could Be a Lever for Improving Seed Quality

As recommended, conventional S fertilization amounts attain 30 kg S ha^-1^ and are applied once at the end of the vegetative rest period in oilseed rape (GS32), when the level of S mineralization in the soil may be low (source Terres Inovia). In our study, we aimed to compare different S amounts and application timings from conventional recommendations and how they affected seed yield and seed quality criteria. It has been shown that in response to S limitation, S remobilization is strongly increased along with enhanced S uptake efficiency *via* an increase in root proliferation and the induction of sulfate transporters in both roots (Hawkesford and De Kok, 2006; Abdallah et al., 2010) and source leaves of oilseed rape (Dubousset et al., 2009; Girondé et al., 2014). Thereby, the hypothesis is that limiting S input at specific stages or fractionating S inputs might allow better S uptake and remobilization, which would lead to better seed protein quality. Our results showed no significant differences in growth performance or S and N uses efficiencies when S inputs was delayed (0+30S_HN) or fractionated (8+22S_HN) (Tables 4, 5). The total N in plants was only significantly higher when S was applied once at bolting (GS32, 30+0S_HN), but this was not observed when applied once at early flowering (GS53, 0+30S_HN). The explanation could be that the amount of S taken up between GS32 and GS53 facilitated greater efficiency in N uptake and assimilation. Oil and protein contents as well as %S in seeds were not reduced or improved by delaying or fractionating the S input (Table 6). Even if delaying S fertilization led to a significant decrease of %N in seeds, this delaying S application improved the seed protein quality by significantly increasing the relative abundance of napin. These observations may lead to reconsideration of the conventional scheme because they demonstrate that fractionated or delayed S fertilizer inputs could (i) meet the requirements in terms of seed yield and quality criteria and (ii) facilitate on time adjustments according to the stage of growth. Coupling this fractionating approach with different levels of N fertilizer might provide additional insights for developing N and S management strategies.

#### Toward New Indicators to Predict Seed Protein Quality

**FIGURE 6:**
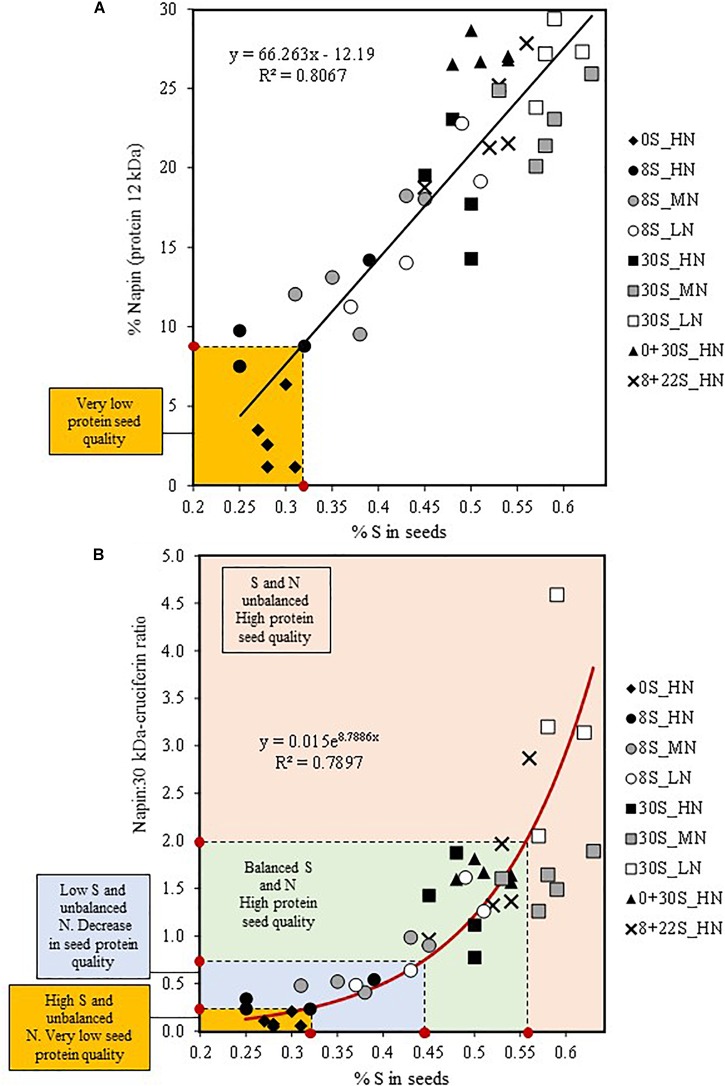
Relationship between the relative S content (% S) and the relative abundance of napin (S-rich SSP of 12 kDa) in mature seeds **(A)** and the relative S content (%S) and the napin:30 kDa-cruciferin ratio **(B)**. Data corresponding to the different S and N fertilization combinations were plotted to determine the correlation (*n* = 41, *p* < 0.0001, RMCE = 3.702).

The protein and oil contents of seeds are crucial factors for farmers and industry (e.g., compensation of farmers based on the percentage of wheat grain protein). However, to date the impact of S and N fertilization on nutritional quality of the grain has rarely been taken into account in winter oilseed rape, which could be detrimental for the baking quality of bread in the case of wheat (Timms et al., 1981; Ortolan and Steel, 2017). In oilseed rape, seeds are used for the production of meal used for livestock feed. The increasing worldwide demand for vegetable protein for human nutrition (vegetarian or vegan diets) has led to a wider search for sources of vegetable protein, thus making oilseed rape proteins interesting alternatives due to their high content of essential S-amino acids (Von Der Haar et al., 2014). Therefore, the protein quality can be associated to the level of S-rich protein content in the seeds, especially like napin. In our study, a strong linear correlation between the relative S content in seed (% of S) and the relative abundance of napin in mature seeds was determined with the data from the nine combinations of S and N fertilization (Figure 6A). Therefore, this relationship suggests that the relative abundance of napin could be predicted by the measurement of S% in seeds. Below a threshold of 0.32% of S in seeds, corresponding to a relative napin abundance of 9%, the protein quality of the seeds is severely reduced (Figure 6A). Our data obtained under controlled conditions have shown the significant relationship between the relative S content and the ratio of napin:30 kDa-cruciferin (Figure 6B). As the napin:30 kDa-cruciferin ratio could be a reliable indicator of the seed protein quality taking into account S and N fertilization, this relationship could provide a new tool for the determination of seed protein quality in oilseed rape according to different N and S managements (Figure 6B). For example, under our experimental conditions, seeds with a napin:30 kDa-cruciferin ratio value below 0.25 corresponded to seeds of very low protein quality and with less than 0.32% of S (Figure 6B). A threshold value at 0.44% of S in seeds should be reached to ensure no deleterious effect of N and/or S availability on seed protein quality. When the napin:30 kDa-cruciferin ratio exceeds 2, which corresponded to S% value higher than 0.56%, protein quality would not be affected but it would indicate an unbalanced management of S and N fertilization (Figure 6B). These indices are interesting new tracks but it will be necessary to test these indices in other trials to verify their relevance and repeatability, or if there is a need to calibrate them for control or field conditions or for specific genotypes. In the perspective of imposing added values according to the markets (e.g., edible oil, vegetable protein), this index (%S in seeds) may provide a relevant tool to direct specific seed lots for different uses according to protein quality levels. Moreover, our results prompt the question about the impact of the variation of napin:30 kDa-cruciferin ratio in terms of nutritional value. Campbell et al. (2016) have mentioned that as oilseed rape proteins are almost exclusively used for animal feed, knowledge of their nutritional value to humans is quite limited. In a randomized cross-over intervention study in humans of an oilseed rape protein isolate containing cruciferin and napin, the Protein Digestibility Corrected Amino Acid Score (PDCAAS) was found to be 0.86, a value close to the soybean protein isolate (Bos et al., 2007). In order to verify the impact of S and N fertilization on the nutritional value of seed proteins in oilseed, further investigations could be scheduled to evaluated PDCAAS on the seeds having contrasted napin:30 kDa-cruciferin ratio.

## Conclusion

Reducing fertilizer inputs while maintaining or even improving seed yield and quality has become an environmental and economic challenge. Our results are a step toward achieving these targets and they provide insights into the joint monitoring of S and N fertilization in oilseed rape. Here we have highlighted the importance of (i) balancing S and N inputs rather than providing a single element in an excessive way and (ii) delaying or fractionating S inputs. In addition, we have demonstrated that the S% and the napin:30 kDa-cruciferin ratio, which could be used as a relevant index for the determination of seed quality, is highly dependent on S/N fertilization in relation to S supply.

## Author Contributions

EP, JT, SB-M, XP, and J-CA were involved in conceptualization of the study. EP, JT, SB-M, and J-CA contributed to the experimental design and tissue sampling. EP, YA, and J-CA carried out the biochemical and elemental analyses. EP and CP performed statistical analyses. EP, JT, SB-M, YA, and J-CA contributed to the interpretation of data and drafting the article. EP, JT, SB-M, and J-CA were involved in revising the manuscript for important intellectual content.

## Conflict of Interest Statement

The authors declare that the research was conducted in the absence of any commercial or financial relationships that could be construed as a potential conflict of interest.

## Footnotes

1 www.bio-rad.com/en-us/product/mini-protean-tgx-stain-free-precast-gels

2 www.bio-rad.com/en-us/product/gel-doc-ez-gel-documentation-system

3 www.bio-rad.com/en-us/product/image-lab-software

## Abbreviations

CRUBnC1: cruciferin poor in S-amino acids
CRU4: cruferin rich in S amino acids
DM: dry matter
GS: growth stage
HI: harvest index
HN: high nitrogen fertilization
LN: low nitrogen fertilization
MN: medium nitrogen fertilization
NHI: nitrogen harvest index
NUE: nitrogen use efficiency
SHI: sulfur harvest index
SSP: seed storage protein
SUE: sulfur use efficiency
U: unit of fertilization (kg of nitrogen or sulfur per ha)
