# Supplementary figures and images for: Seed Yield Components and Seed Quality of Oilseed Rape Are Impacted by Sulfur Fertilization and Its Interactions With Nitrogen Fertilization
Source: Front Plant Sci. 2019 Apr 16;10:458. doi: 10.3389/fpls.2019.00458 (PMC6477675; doi:10.3389/fpls.2019.00458)

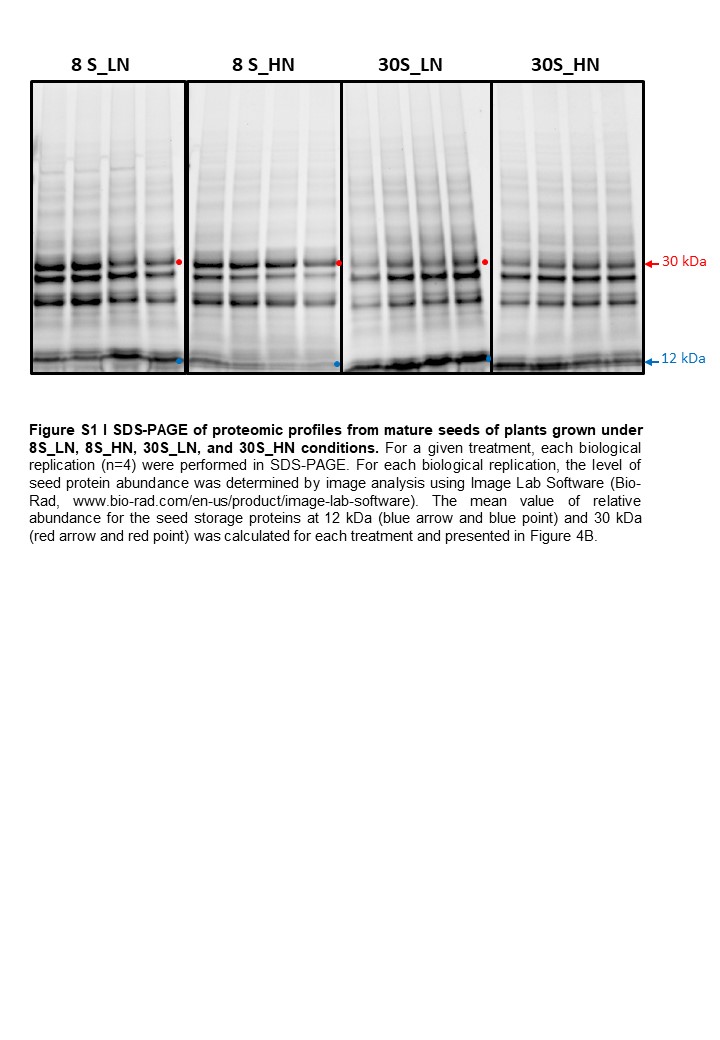

Supplement: Supplementary file 1 [file Image_1.JPEG]
